# Cardiac Calcified Amorphous Tumor of the Mitral Valve Presenting as Transient Ischemic Attack

**DOI:** 10.1155/2017/2376096

**Published:** 2017-01-17

**Authors:** Mohammad Abbasi Teshnizi, Atefeh Ghorbanzadeh, Nahid Zirak, Babak Manafi, Aliasghar Moeinipour

**Affiliations:** ^1^Department of Cardiac Surgery, Imam Reza Hospital, Atherosclerosis Prevention Research Center, Faculty of Medical Science, Mashhad University of Medical Sciences, Mashhad, Iran; ^2^Student Research Committee, Mashhad University of Medical Sciences, Mashhad, Iran; ^3^Faculty of Medicine, Mashhad University of Medical Sciences, Mashhad, Iran; ^4^Faculty of Medicine, University of Medical Sciences, Hamedan, Iran

## Abstract

Cardiac calcified amorphous tumors (CATs) are an extremely rare nonneoplastic intracardiac masses. They have been reported in the literature in only a few cases. Thus, the incidence, pathogenesis, and best approach to the treatment are not certain. We report a case of CATs on the atrial surface of the anterior mitral valve leaflet in a 37-year-old female who was diagnosed by histopathological examination after surgical removal.

## 1. Introduction

Calcified amorphous tumors (CATs) of the heart are an exceedingly rare nonneoplastic intracardiac mass that was originally described in 1997 by Reynolds et al. [1]. The clinical features of cardiac CAT are usually like the other cardiac masses which include the symptoms related to obstruction or embolization such as dyspnea and syncope. Thus, it may be misdiagnosed with other cardiac tumors [2, 3]. Accurate diagnosis of a cardiac mass is often made on surgical excision and histopathological examination [1–3]. Currently, only a few cases of cardiac CATs have been reported in the literature. The incidence, pathogenesis, and best approach to the treatment are not certain. Herein, we describe a case of cardiac CATs originating from the mitral valve in a 37-year-old woman revealed by the transient ischemic attack (TIA).

## 2. Case Presentation

**Figure 1:**
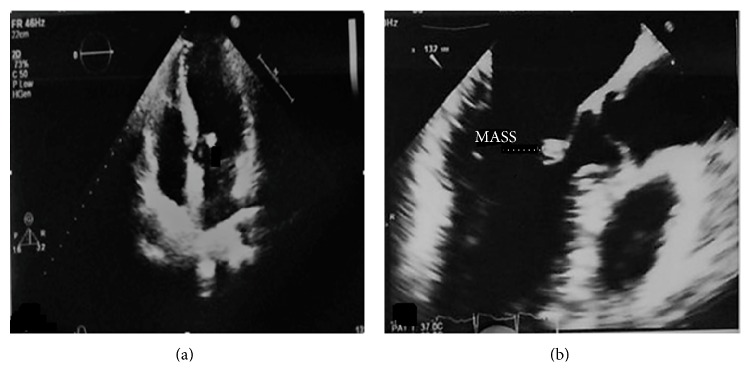
Transesophageal echocardiography showing an echogenic, round, and mobile mass, attached to the atrial surface of the anterior mitral valve leaflet by a short pedicle, measuring 5 × 5 mm.

**Figure 2:**
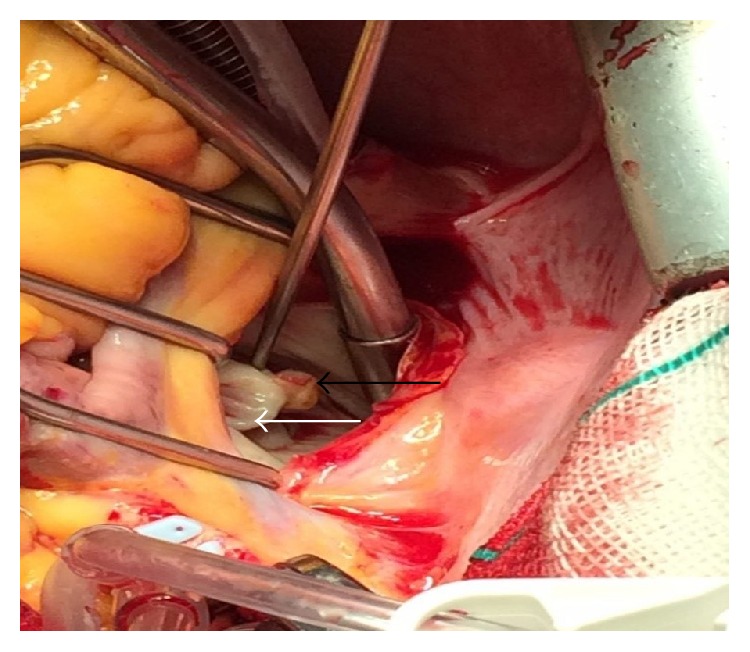
Intraoperative findings evidencing calcified tumor (black arrow) attached to the atrial surface of the anterior leaflet (white arrow) of the mitral valve.

A 37-year-old woman sought neurological assistance after an episode of TIA manifesting predominantly as left hemiparesis of 5-minute duration. At that time, she had no abnormalities on examination of the cranial nerves, and brain computed tomography (CT) scan findings were normal. She was referred to a cardiologist to be evaluated for TIA. The physical examination and routine blood laboratory investigations results were normal. Her past medical history showed hypertension (HTN) and hyperlipidemia (HLP) which both were under control. During further clinical assessments, transesophageal echocardiography (TEE) was performed, which revealed an echogenic, round, and mobile mass measuring 5 × 5 mm in diameter attached by a short pedicle on the atrial surface of the anterior mitral valve leaflet (Figure 1). There was mild to moderate regurgitation of the mitral valve. The other valves and cardiac structures were normal. Left ventricular ejection fraction (EF) was 60%. The patient was given anticoagulant therapy and referred for surgery with clinical suspicion of a papillary fibroblastoma or other primary cardiac tumors. At operation, after a median sternotomy, cardiopulmonary bypass via aortobicaval cannulation was established. An incision was made in the left atrium, and a soft, firm, and friable mass was founded on the atrial surface of the anterior leaflet of the mitral valve which was easily removed (Figure 2). The anterior leaflet was repaired with an autologous pericardium patch. The patient was warmed and weaned from cardiopulmonary without difficulty.

**Figure 3:**
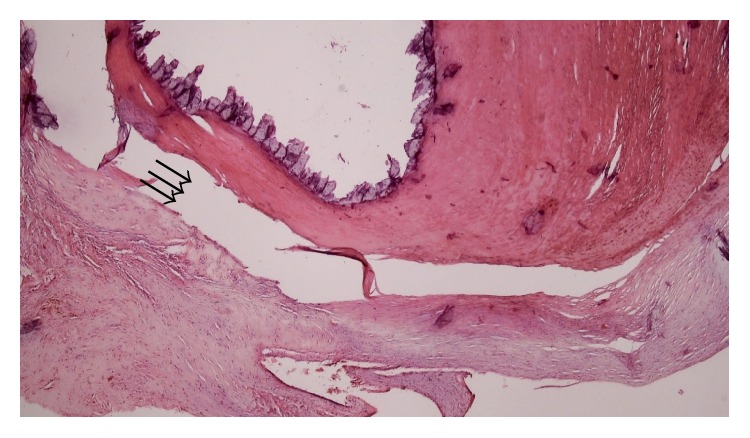
Histological examination showed dense calcification (shredded due to no decalcification) (arrows) in a background of amorphous degenerating fibrinous material (hematoxylin and eosin stain; ×40).

Histopathological evaluation of the resected tumor showed a dense calcification (shredded due to no decalcification) in a background of amorphous degenerating fibrinous material (Figure 3) and according to the clinical and histological features, a diagnosis of cardiac CAT was provided. The postoperative course was uneventful, and the patient was discharged from the hospital 4 days after the surgery without any complications.

## 3. Discussion

Intracardiac masses have been classified as neoplastic and nonneoplastic lesions. Cardiac tumors are also divided into benign (about 75%) and malignant neoplasms in which more than half of the benign tumors are myxomas [4]. Cardiac CAT which recently is described as a distinct pathological entity is extremely rare cardiac benign nonneoplasm [1]. The clinical manifestations of CAT are not specific and similar to those of other cardiac masses, often related to embolization or flow obstruction of the calcified fragments. Thus, differentiation between various types of cardiac masses, such as myxomas or fibroma, calcified thrombi, emboli, vegetation, and other intracardiac tumors may be difficult. The most common symptoms of these masses are progressive dyspnea, chest pain, syncope, and symptoms related to embolism [2, 3, 5]. Rarely, they may also present with cardiac arrhythmias such as ventricular tachycardia [3]. Further, mobile CATs have a higher chance of embolic event than immobile lesions. Even though modern cardiac imaging modalities can help to narrow down the differential diagnosis, surgical excision of the lesion and histological examination are necessary for definitive diagnosis [6]. Histological features of cardiac CATs consist of nodular calcium deposits within a background of fibrin or amorphous fibrillar material. Although they may occur in any chamber of the heart, they are found most commonly in the left ventricle (31.25%) and mitral valve (25%). Approximately 12.5% of cases of the cardiac CATs originated from the right atrium [7]. In our case, cardiac CATs were mobile and found in the mitral valve with initial presentation of TIA. The average age at diagnosis is 51 years with a range from 18 to 78 years. Prevalence is slightly more frequently in females [7]. The patient in our report was a 37-year-old woman. Although the exact pathogenesis of cardiac CAT is not determined, some authors believe the hypothesis that cardiac CAT is an organized and calcified mural thrombus [8]. Kawata et al. also suggested that disorders of calcium-phosphorus metabolism in patients with renal dysfunction can be potential causes of CAT [9]. On the other hand, the lack of such predisposing conditions in other cases, like the current case, proposes that thrombosis may not be the only pathogenetic mechanism and also other various mechanisms can be involved. Surgical excision is mandatory because of the risk of complications or embolization and also for accurate diagnosis. Recurrence and fatal outcomes of cardiac CATs have rarely been reported after surgical resection, and, consequently, long-term follow-up with cardiac imaging studies, especially in patients with incomplete resection, is required [6, 10]. In our case, the initial diagnosis was thought to be fibroblastoma or other primary cardiac tumors, but after resection of the tumor and histopathological evaluation, the diagnosis of CATs was confirmed. To summarize, we reported a case of CATs on the atrial surface of the anterior mitral valve leaflet in a 37-year-old female which was confirmed by histopathological examination after surgical removal.

## 4. Conclusion

The CATs of the heart are extremely rare nonneoplastic intracardiac mass and accurate diagnosis of a cardiac mass is often made on surgical excision and histological examination for the best management of them.
